# Shifts in the Distribution Range and Niche Dynamics of the Globally Threatened Western Tragopan (*Tragopan melanocephalus*) Due to Climate Change and Human Population Pressure

**DOI:** 10.3390/biology12071015

**Published:** 2023-07-17

**Authors:** Muhammad Azhar Jameel, Muhammad Sajid Nadeem, Shiekh Marifatul Haq, Iqra Mubeen, Arifa Shabbir, Shahzad Aslam, Riyaz Ahmad, Abdel-Rhman Z. Gaafar, Bander M. A. Al-Munqedhi, Rainer W. Bussmann

**Affiliations:** 1 Department of Zoology, Wildlife & Fisheries, PMAS-Arid Agriculture University, Rawalpindi 46300, Pakistan; azherjamil812@gmail.com (M.A.J.); sajidnm@uaar.edu.pk (M.S.N.); shahzad_zoologist@yahoo.com (S.A.); 2 Department of Ethnobotany, Institute of Botany, Ilia State University, 0162 Tbilisi, Georgia; rainer.bussmann@iliauni.edu.ge; 3 Department of Zoology, Government College University, Lahore 54300, Pakistan; iqramubeen367@gmail.com (I.M.); arifashabbirkhn@gmail.com (A.S.); 4 National Center for Wildlife, Riyadh 11575, Saudi Arabia; riyaz.cf@gmail.com; 5 Department of Botany and Microbiology, College of Science, King Saud University, Riyadh 11451, Saudi Arabia; agaafar@ksu.edu.sa; 6 Department of Botany, Institute of Life Sciences, State Museum of Natural History, 76133 Karlsruhe, Germany

**Keywords:** *Tragopan melanocephalus*, western Himalayas, climate change, anthropogenic pressure, species recovery

## Abstract

**Simple Summary:**

Given the potential impact of climate change on the distributions of endemic species, it is critical to implement species recovery and habitat management measures to protect threatened species. This study used MaxEnt modeling to assess the species’ habitat suitability in varying climate scenarios in the Western Himalayas and identified ten influential drivers. Our field-based observations of *Tragopan melanocephalus* show that the species typically lives at elevations between 1850 and 3800 m, which is consistent with the species’ reported affinity for extremely high elevations between 1500 and 4000 m above sea level. Our findings reveal that areas with high and moderate suitability for the species are patchily distributed throughout the Western Himalayas, ranging from northeastern Pakistan to central Himachal Pradesh and Uttarakhand. Moreover, there are continuous strips of highly suitable habitats along the Pakistan–Afghanistan border, in the Kashmir region, and in the Annapurna region of Uttarakhand. The study observed that the Western Tragopan’s habitat suitability may increase under future climate change scenarios, but additional research is needed to avert population collapses and identify other potential drivers of local extinction occurrences. To support increased biodiversity and lower risks under current and anticipated future climatic conditions, it is advised that the suitable areas identified be designated as nature reserves. According to the study’s findings, a more effective wildlife management strategy might significantly help with the reintroduction of the *Tragopan melanocephalus* population into its suitable habitats in western Himalaya, thereby advancing the global objectives set for the UN Decade of Ecosystem Restoration targets (2021–2030).

**Abstract:**

The impact of a changing climate, particularly global warming, often harms the distribution of pheasants, particularly those with limited endemic ranges. To effectively create plans of action aimed at conserving species facing threats such as the Western Tragopan, (*Tragopan melanocephalus;* Gray, 1829; Galliformes, found in the western Himalayas), it is crucial to understand how future distributions may be affected by anticipated climate change. This study utilized MaxEnt modeling to assess how suitable the habitat of the targeted species is likely to be under different climate scenarios. While similar studies have been conducted regionally, there has been no research on this particular endemic animal species found in the western Himalayas throughout the entire distribution range. The study utilized a total of 200 occurrence points; 19 bioclimatic, four anthropogenic, three topographic, and a vegetation variable were also used. To determine the most fitting model, species distribution modeling (SDM) was employed, and the MaxEnt calibration and optimization techniques were utilized. Data for projected climate scenarios of the 2050s and 2070s were obtained from SSPs 245 and SSPs 585. Among all the variables analyzed; aspect, precipitation of coldest quarter, mean diurnal range, enhanced vegetation index, precipitation of driest month, temperature seasonality, annual precipitation, human footprint, precipitation of driest quarter, and temperature annual range were recognized as the most influential drivers, in that order. The predicted scenarios had high accuracy values (AUC-ROC > 0.9). Based on the feedback provided by the inhabitants, it was observed that the livability of the selected species could potentially rise (between 3.7 to 13%) in all projected scenarios of climate change, because this species is relocating towards the northern regions of the elevation gradient, which is farther from the residential areas, and their habitats are shrinking. The suitable habitats of the *Tragopan melanocephalus* in the Himalayan region will move significantly by 725 m upwards, because of predicted climate change. However, the fact that the species is considered extinct in most areas and only found in small patches suggests that further research is required to avert a further population decline and delineate the reasons leading to the regional extinction of the species. The results of this study can serve as a foundation for devising conservation strategies for *Tragopan melanocephalus* under the changing climate and provide a framework for subsequent surveillance efforts aimed at protecting the species.

## 1. Introduction

In an era of global climatic change, the distributions of endemic species change at accelerating rates [1]. Such changes are already altering the composition of ecological communities, but they go beyond natural system conservation [2]. A recent worldwide report has estimated that approximately one million plant and animal species are currently at risk of extinction [3]. This unparalleled loss of biodiversity jeopardizes most of the United Nations’ sustainable development goals, including the elimination of poverty and associated environmental issues [4]. Hence, the foremost environmental priorities of the twenty-first century are the conservation of endangered species worldwide and the rehabilitation of damaged ecosystems [5].

One of the main causes of the loss of biodiversity worldwide is unsustainable human activities, resulting in habitat destruction and overexploitation of endemic species [6,7]. Many species are anticipated to alter their present distribution ranges in reaction to climate change to adapt to the altered environmental conditions [8]. While all ecosystems are affected by climate change, high-mountain ecosystems, such as those in the Western Himalayan range, are thought to be more susceptible to warming [9,10]. By the end of the twenty-first century, suitable habitats for several high-mountain animal species may be substantially diminished or even gone, especially in areas where global warming and precipitation decline are occurring together [11]. The biodiversity will be affected by climate change, which may cause species to decline or even go extinct [12,13]. The impact of climate change on biodiversity will be multifaceted, with direct effects arising from fluctuations in temperature and precipitation regimes, as reported by Huang et al. [14], and indirect effects resulting from alterations in permafrost patterns, disturbance dynamics, and shifts in biotic interaction, as noted by Schmeller et al. in his study [15].

In response, the scientific and practitioner communities have amplified their efforts in researching and planning management interventions to support the adaptation of nature to climate change [16]. Prioritizing disturbed landscapes that can support biodiversity protection is essential for conducting recovery initiatives, especially for endemic species that are more susceptible to extinction [17]. In the pursuit of species recovery, conservation managers may encounter difficulties in determining the optimal locations and methods for recovery, especially given the current climate change scenario [18]. Species distribution modeling (SDM) is a promising approach for formulating recovery strategies for endangered endemic species in regions with rich biodiversity. This technique enables the identification of suitable habitats and the prediction of range shifts under climate change projections [19]. The SDM approaches, based on the niche conservatism hypothesis, use data on species distribution combined with climatic and other environmental variables to predict the distribution of species along spatiotemporal gradients [20]. Thus, integrating macro-spatial SDM insights with local-scale ecology can help develop successful recovery plans for species that are threatened globally [21].

The Western Tragopan (WT) is an endemic species found in the western Himalayas, ranging from the Indus-Kohistan region in northern Pakistan to Uttarakhand in northwest India through Kashmir [22]. According to Shah et al. [23], the IUCN Red List has classified this galliform species as “Vulnerable” due to its dwindling and widely dispersed population, which is continuously declining in numbers and becoming fragmented as a result of deforestation, browsing of understory shrubs by livestock, and tree lopping for fodder and fuelwood collection, as well as illegal hunting and habitat degradation, all of which pose a significant threat to its already limited habitat and declining population [24]. In the Himalayas, a rapidly changing climate is expected to increase extinction risks, especially for endemic vulnerable species, necessitating the inclusion of climate change-related risks in conservation plans and species recovery approaches. This study will estimate how the WT range might change concerning various future climate change scenarios and examine WT’s current suitable habitats across its entire distribution range.

The objective of this study was to address recovery issues related to the Western Tragopan by answering the following questions: (i) What environmental factors are critical; what are the ideal microhabitats for the species in the western Himalayas under the current and projected scenarios of climate change? (ii) How will the distribution range of *Tragopan melanocephalus* be impacted by various future climate change scenarios? (iii) How can the predictions derived from species distribution models (SDMs) be utilized to guide the recovery efforts of the Western Tragopan in the study area? Finally, we aim to offer practical recommendations for the conservation and recovery of the species in the region based on field observations.

## 2. Materials and Methods

### 2.1. Study Area

**Figure 1:**
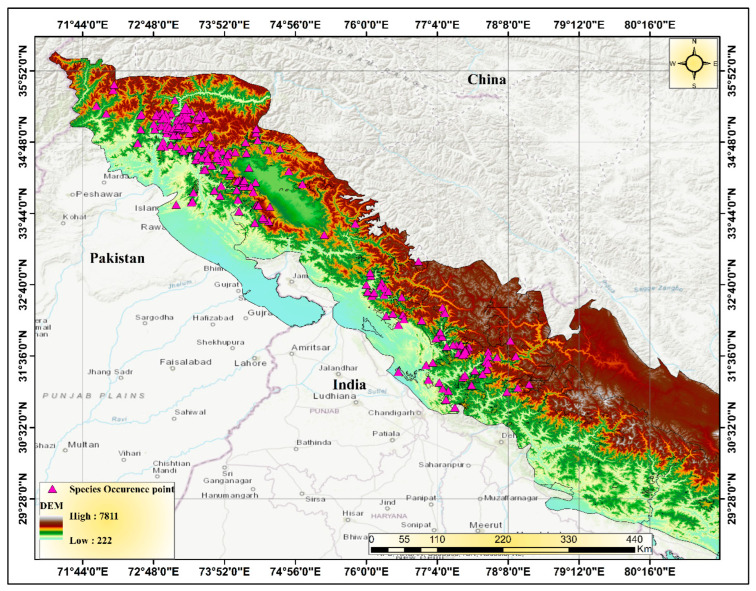
Digital elevation modeling (DEM) of the study area in the Western Himalayas in South Asia.

The Himalayas, known as the “Abode of Snow”, are a relatively young mountain range that acts as a natural barrier between the Indo-Gangetic Plain and the Tibetan Plateau. Extending approximately 2400 km from Afghanistan in the west to Burma in the east, this majestic range spans latitudes 27–36° N and longitudes 72–91° E, serving as a crucial link between the Near East, Central Asia, and East Asia. The Himalayan range is shared by five countries—Nepal, India, China (Tibet), Bhutan, and Pakistan—with the majority of it located within the first three countries [25]. The Western Himalaya refers to the western part of the Himalayan mountain range and encompasses an area of approximately 130,000 km^2^ (Figure 1). It stretches from Badakhshan in Afghanistan, through northern Pakistan (including Khyber Pakhtunkhwa and the Jammu and Kashmir region), to North India (including Jammu and Kashmir, Ladakh, and Himachal Pradesh) and Nepal [26,27,28].

### 2.2. Species Presence Data

The presence data of *Tragopan melanocephalus* in the study area was collected using a combination of methods. To collect information on species distribution points (presence points), six years of field surveys were carried out and published data were gathered from the literature and the GBIF (Global Biodiversity Information Facility) database from 1864 to 2019. Duplicate records were eliminated to avoid the overrepresentation of certain areas. The selection of presence points was conducted with careful consideration to prevent spatial pseudo-replication. The resolution of the raster data, which was 2.5 arc-minutes, guided the process. Only GPS locations with an accuracy of 15 m were chosen and filtered to ensure that only one presence point was retained within each 5 km^2^ pixel. This approach was adopted to avoid any potential bias that may arise from having multiple presence points in close proximity to each other. This approach is common in species distribution modeling to ensure that the data used are not biased and the species distribution in the study area is represented with accuracy.

**Figure 2:**
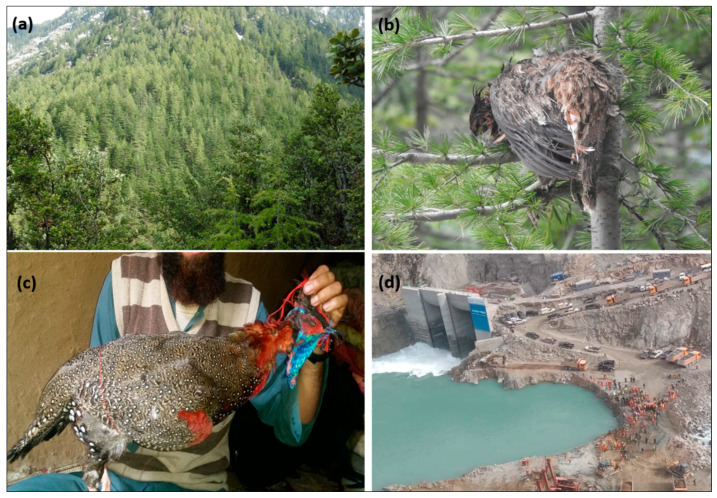
Photographs during our field surveys depicting (**a**) the natural environment of *Tragopan melanocephalus*, (**b**) a female western Tragopan in its ecological zone, (**c**) a male *Tragopan melanocephalus* that had been hunted by hunters in Palas valley, and (**d**) an extensive infrastructure development project located deep inside the species’ natural habitat.

Our research team conducted several field surveys from 2016 to 2022 to document the presence points of *Tragopan melanocephalus* in the Western Himalayas. We used binoculars (8 × 40) and spotting scopes (15 × 45) to observe the species from different trails and viewpoints in their potential habitat. For every sighting, we recorded the habitat characteristics, including the proximity to the nearest cliff and the degree of slope ruggedness. We also recorded the location and elevation using a handheld GPS (Global Positioning System) device. In addition, we also kept a watch out for illicit activities including habitat destruction, illegal hunting and poaching, and natural disturbance throughout our field surveys (Figure 2). To gather more information about the possible presence of the Western Tragopan, and illegal activities in potential habitats, we deployed a questionnaire survey that we administered to knowledgeable local personnel and staff from different departments. We assessed their knowledge of the species through discussions and interactions with them. We obtained a total of 200 distinct presence points of the *Tragopan melanocephalus* to develop the Species Distribution Model (SDM).

### 2.3. Environmental Data Collection and Variable Selection

The study acquired 19 bioclimatic factors with a resolution of 2.5 arc-minutes and elevation information with a resolution of 30 arc-seconds from the WorldClim website (ver. 2.1) (www.worldclim.org, accessed on 15 March 2023) (Table 1). Topographic input data comprising altitude, slope gradient, and aspect were chosen and extracted as variable layers from the ArcGIS 10.5 software and used to apply spatial analysis tools for creating a Digital Elevation Model (DEM). We incorporated four anthropogenic variables to act as proxies for human impact in addition to environmental factors. These variables were Land cover, Road proximity, Population density, and the Human footprint (HFP), which measures human perturbation. The HFP map, which was sourced from the Socio-Economic Data and Applications Centre (http://sedac.ciesin.columbia.edu. accessed on 5 April 2022), integrates worldwide data layers associated with factors that potentially impact ecosystems, such as human population distribution, urbanization, navigable rivers, roads, and diverse agricultural land uses. The scale of the HFP layer ranges from 0, which represents areas that are near natural or pristine, to 50, which suggests regions that are severely degraded.

The Global Land Cover data included nine categories: forest, shrubland, savannah, grassland, cropland/natural vegetation, wetland, urban, snow/ice, and barren/sparsely vegetated areas. The source of the data was the International Geosphere-Biosphere Program (MODIS Global Land Cover Classification v2, http://www.modis.bu.edu/landcover. accessed on 5 April 2022). The population density was gathered from Oak Ridge National Laboratory (http://www.ornl.gov/sci/landscan. accessed on 4 April 2022) as a separate layer because it has been demonstrated to significantly affect the dispersion of invasive species. For the vegetation variable, we utilized the enhanced vegetation index (EVI) of the MODIS products (MOD13A3) and followed the same variability in climatic variables for vegetation, namely, mean annual EVI and EVI seasonality. We chose to employ EVI (Enhanced Vegetation Index) rather than the more widely used NDVI (Normalized Difference Vegetation Index) due to its capacity to decrease canopy background fluctuation and preserve sensitivity in areas with dense vegetation. EVI is also better able to deal with lingering smoke and sub-pixel cloud contamination in the atmosphere. EVI is Terra MODIS ready-to-use files (MOD11 and MOD13, respectively) that can be downloaded from GLOVIS (http://glovis.usgs.gov/ accessed on 4 April 2022). For future simulations, we acquired two Shared Socioeconomic Pathways (SSPs)—SSPs 245 and SSPs 585—from the Coupled Model Intercomparing Project, Phase 6 (CMIP6) for two distinct timeframes: the 2050s (2041–2060) and the 2070s (2061–2080). To perform these simulations, we utilized the Global Climate Model of BCC-CSM2-MR, which has a resolution of 2.5 arc-min.

Various factors, such as precipitation, temperature, geographical barriers, geological formations, and other biological variables, collectively affect the distribution of the Western Tragopan [29,30]. To evaluate which environmental factors affect the species’ distribution, our model employed 19 bioclimatic, four anthropogenic (human footprint, road proximity, land cover, and human population density), and three biophysical (aspect, elevation, and slope) variables along with vegetation.

This study used a two-step procedure to guarantee independence and omit spatially linked data items. First, a preliminary model with default settings was implemented to ascertain the contribution of each variable. We set a threshold of >1% to filter out variables that did not meet the criterion. Secondly, in order to locate and eliminate any potential spatial association, we then assessed the remaining variables (above the contribution threshold) for pairwise Pearson’s association (r). To reduce the number of variables further, a threshold value (r ≥ ±0.8) was employed. If two variables had an r value above this threshold, the one with the lesser contribution was omitted [29,31,32,33,34,35,36].

### 2.4. Preliminary Variables Processing

**Figure 3:**
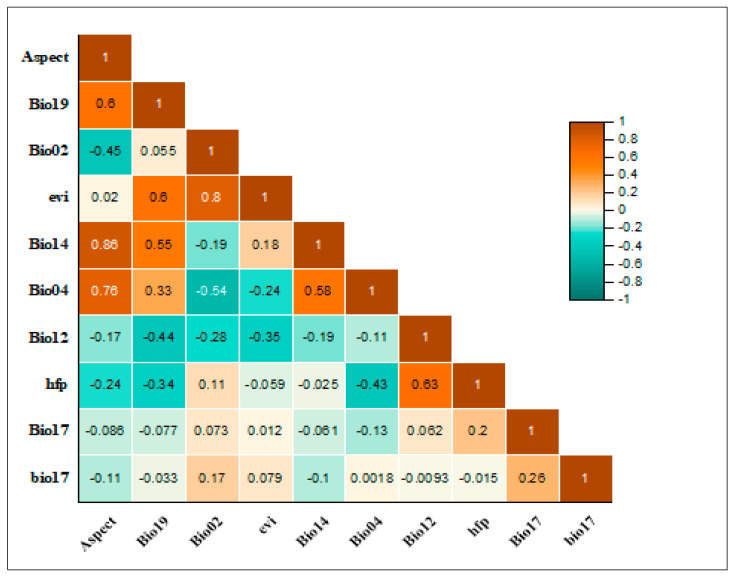
A heat map of Pearson correlations displaying the pairwise correlations (with a threshold of r = ±0.8) between the climatic and biophysical variables used in the distribution modeling of the *Tragopan melanocephalus*.

After applying the contribution and Pearson’s correlation coefficient threshold, 10 important bioclimatic and topographic variables were identified. These variables include aspect, precipitation of coldest quarter, mean diurnal range, enhanced vegetation index, precipitation of driest quarter, temperature seasonality, annual precipitation, human footprint, precipitation of driest quarter, and temperature annual range [37]. The pairwise correlation between these selected variables can be seen in Figure 3.

### 2.5. Model Calibration and Optimization

In species distribution modeling (SDM), calibrating and optimizing MaxEnt prediction models is crucial for selecting the best model. This is often achieved by tuning the model with various regularization multiplier (RM) values and feature classes (FC) to improve prediction reliability and prevent overfitting. To identify the optimal MaxEnt model settings, threshold-dependent evaluation metrics (i.e., omission rate) were used to improve model transferability. The study targeted multiple combinations of eight RM values (ranging from 1 to 4 with a 0.5 interval) and six FCs (L, LQ, H, LQH, LQHP, and LQHPT where L = Linear, Q = Quadratic, H = Hinge, P = Product, T = Threshold) [35,38].

To generate the bias file for our model, we utilized the ENMEval package in the R programming environment. The MaxEnt version 3.4.4 was employed to assess the data and make predictions regarding the optimal habitats for *Tragopan melanocephalus* within our study area, following established methodologies [34,39,40,41]. The MaxEnt model, which is rooted in ecological niche theory and incorporates information from presence data, allows us to estimate the potential distribution of the target species in our research region [37,42]. MaxEnt software is recognized as one of the most advanced and promising methods for species distribution modeling, as it excels in accurately identifying the most suitable habitats for a given species based on available presence data [38,40,43,44]. Due to its superior prediction accuracy, MaxEnt has become the preferred approach over other methods [42,43,45,46].

To investigate the relationship between environmental conditions and wildlife distributions, we utilized MaxEnt, a reliable machine-learning technique for species distribution modeling [47,48]. Our goal was to determine a link between climatic variables and the occurrences of the species. The 10th percentile presence probability of the species, a 10-fold cross-validation approach, a complementary log-log (clog-log) output format, 10,000 background points, 10 repeat runs, 500 iterations, response curve generation, and an analysis of Jackknife importance in all final optimized SDMs were some of the MaxEnt configurations we used to improve the model’s accuracy and performance.

### 2.6. Reclassification of Predictions and Model Evaluation

The receiver-operator characteristic (ROC) curve’s area under the curve (AUC) measurements were used to assess how well the optimized SDMs performed. Better model prediction accuracy is indicated by a high AUC-ROC value; a good score is one of 0.9 or higher [34,40,43,44,46,49,50]. The AUC score serves as an indicator of the model’s performance in terms of fitting the test data and its ability to discriminate between different variations in species distribution under potential future climate conditions [34,40,44]. An AUC value of 0.5 suggests that the model’s performance is no better than random chance, while a value closer to 1.0 indicates that the model performs better than chance [51]. The AUC score provides valuable insights into the accuracy and reliability of the model’s predictions. The MaxEnt prediction output for the possible Western Tragopan habitat suitability ranged from 0 to 1 and was classified into five levels of suitability: not suitable habitat (NS) (0–0.2), low suitability habitat (LS) (0.21–0.4), moderately suitable habitat (MS) (0.41–0.6), highly suitable habitat (HS) (0.61–0.80), and very highly suitable (VHS) (0.81–1) [40,45,52].

## 3. Results

### 3.1. Model Evaluation

**Figure 4:**
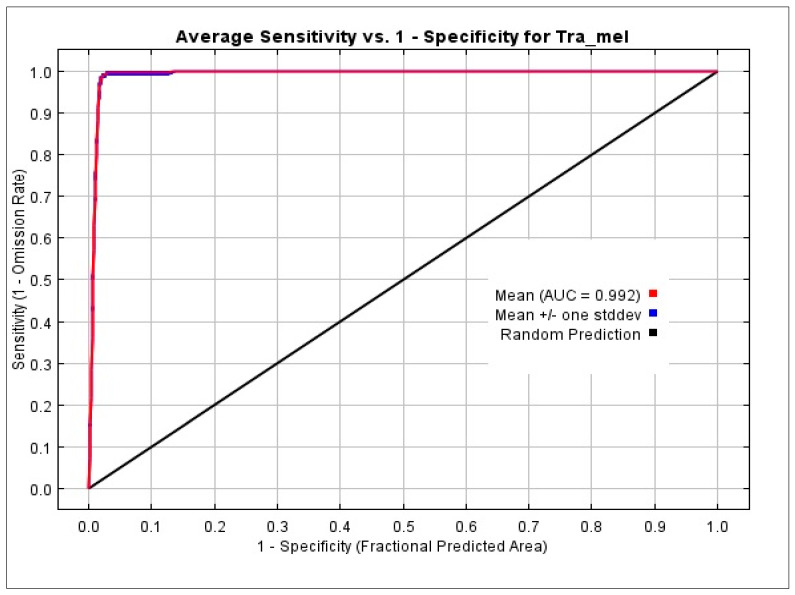
ROC prediction using the MaxEnt model. The model’s precision was 0.992.

We ran MaxEnt for 10 replications and averaged the results to generate a single model prediction. This is a good practice as it helps to reduce the potential influence of random variation in the model. Response curves can visually illustrate the correlation between environmental variables and the estimated probability of species occurrence. These curves can help to identify which variables have the strongest influence on the species distribution and can aid in understanding the ecological factors driving the species distribution. The AUC (area under the curve) graph is a popular tool for assessing how well species distribution models function. The area under the curve (AUC), which measures the model’s capacity to distinguish between the locations of a species presence and absence, is used to assess the model’s performance. The next step is to establish suitability inferences and identify prospective migration paths using the average model. The best candidate model in this investigation, with an AUC score of 0.992, was created using LQH as FCs and an RM value of 1.5. We used MaxEnt to generate a predictive habitat suitability score for the *Tragopan melanocephalus* in the Western Himalayan region. This score ranged from 0 to 0.99, indicating the predicted suitability of different areas for the species. The MaxEnt model’s predictive performance is measured by the AUC value, which shows how well it can distinguish between sites that have the species and those that do not. The AUC ranges between 0.5 (no better than random) and 1.0 (perfect discrimination). While the AUC is a helpful indicator of model performance, it should not be the only factor considered when assessing the model. Our findings suggest that the MaxEnt model created a ROC (Receiver Operating Characteristic) curve automatically (Figure 4). The genuine positive rate (sensitivity) vs. the false positive rate (1-specificity) for various anticipated probability thresholds is plotted on the receiver operating characteristic (ROC) curve. This shows how the model can identify real positives with accuracy while minimizing false positives. Our curve analysis demonstrated that the average AUC value of the model was 0.992, indicating a significantly higher performance compared to the AUC of the random prediction model (0.5). This shows that the predictions made by the model were very accurate and closely matched the actual range of the species.

### 3.2. Elucidating the Ecological Factors Underlying the Spatial Distribution Patterns

The variables that contribute most to *Tragopan melanocephalus’* habitat appropriateness include aspect, precipitation of coldest quarter; mean diurnal range, enhanced vegetation index, precipitation of driest quarter, and temperature seasonality. The factors that had a comparatively small impact on the SDMs of *Tragopan melanocephalus* were annual precipitation, precipitation of the driest quarter, human footprint, and temperature annual range (Table 2).

**Figure 5:**
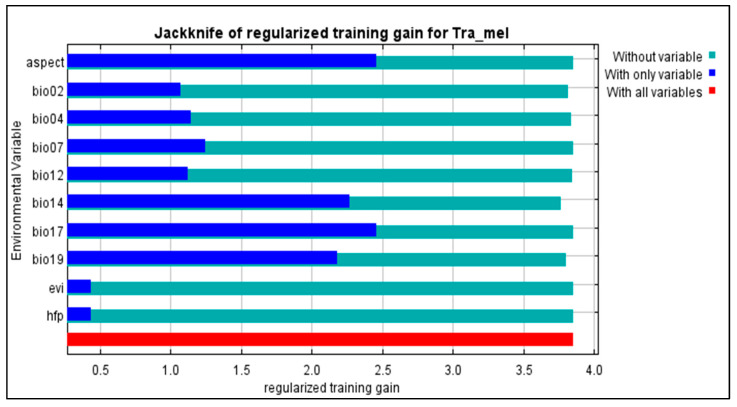
MaxEnt Modeling of *Tragopan Melanocephalus*: Evaluating the Predictive Power of Environmental Variables using Jackknife Regularized Training Gain.

**Figure 6:**
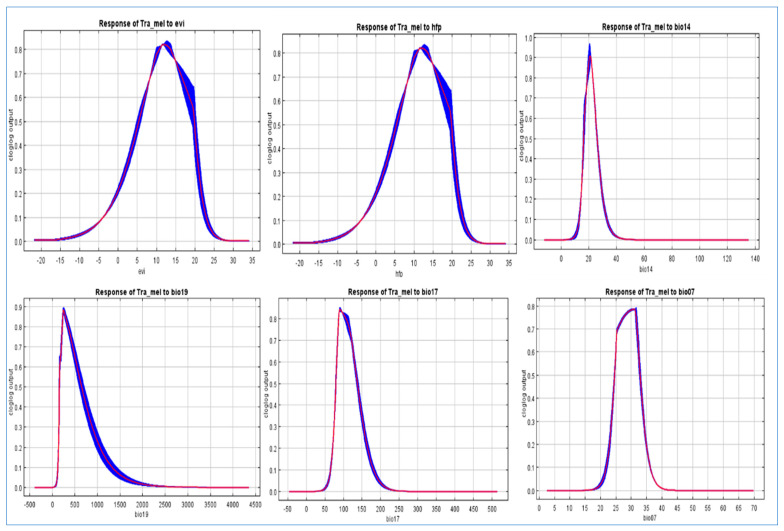
Variable Response Curves (ROC) of the Distribution of *Tragopan Melanocephalus*: Analysis of Enhanced Vegetation Index (EVI), Human Footprint (HFP), and Key Climatic Factors (Bio14, Bio17, Bio19, Bio07).

The findings suggest that the chosen variables aptly captured the present distribution of *Tragopan melanocephalus*. Specifically, the Jackknife test revealed that the aspect and precipitation of the coldest quarter (bio19) were particularly influential in shaping the species’ distribution, contributing to 36.5% and 34.7% of the variance explained by the MaxEnt model, respectively (Figure 5). Due to their high contribution rates, we conducted a separate analysis of the influence of aspect and bio19 as individual factors. The probability of *Tragopan melanocephalus* presence initially increased and then stabilized as annual precipitation increased, as shown in Figure 6.

**Figure 7:**
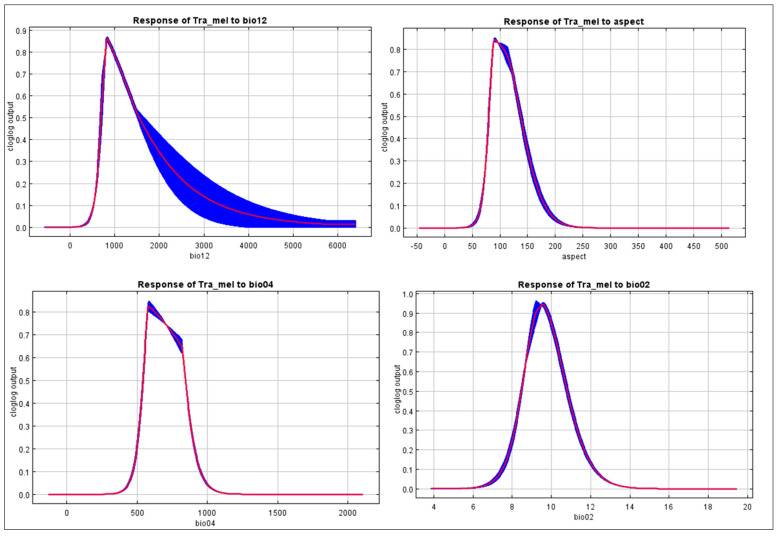
The response curve of highly contributed variable in the distribution of *Tragopan melanocephalus*. Annual precipitation (bio12), aspect, temperature seasonality (bio04), and mean diurnal range (bio02).

Furthermore, as annual precipitation increased, there was an initial increase in the chance of *Tragopan melanocephalus* presence, which then stabilized. This suggests that annual precipitation may have a complex relationship with the presence of the species, and further research may be necessary to fully understand this relationship. When the annual precipitation exceeded 250 mm, the probability of encountering *Tragopan melanocephalus* was above 0.6, indicating the presence of highly suitable areas for the species. The peak chance of occurrence was at approximately 500 mm, with a likelihood of around 0.7. The suitable habitats with high probability had an aspect between 60–100 degrees, as shown in Figure 7.

### 3.3. Current Distribution

**Figure 8:**
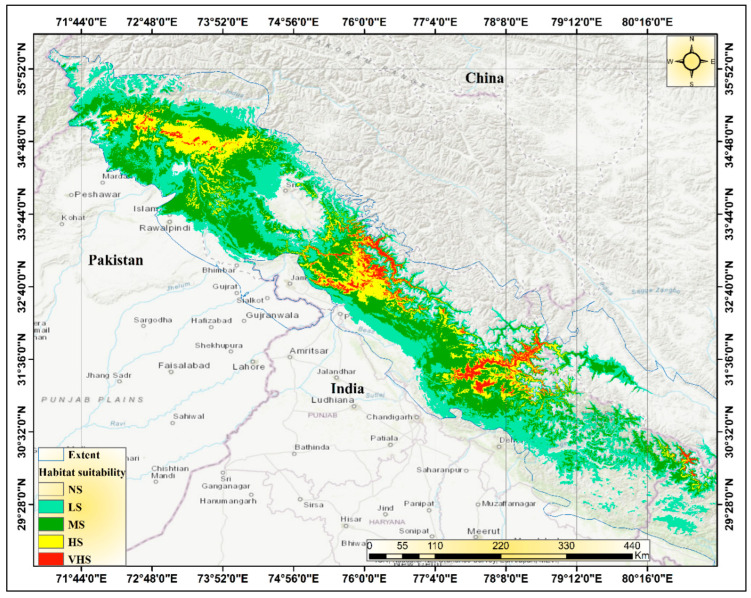
MaxEnt prediction map of *Tragopan melanocephalus’* possible habitat suitability classification in the study region under current climatic conditions (the 1970s–2000s).

The Himalayan range is indeed a vast and geographically diverse region, spanning seven Asian countries including India, Nepal, and Pakistan. With an area of approximately 2500 km^2^, it covers a significant portion of the Indian subcontinent, including 11 states and the Union Territory of Jammu and Kashmir. The Western Himalayas include several mountain ranges of India, including the Zanskar Range, Pir Panjal Range, Dhauladhar Range, and western parts of the Sivalik Range and the western Himalayas. The Zanskar Range is a sub-range of the Great Himalayas, located in the Indian state of Jammu and Kashmir. The Pir Panjal Range is a subrange of the Himalayas that runs from central Afghanistan to northern Pakistan and northern India. The Dhauladhar Range is a subrange of the Himalayas that runs from the Indian state of Himachal Pradesh to the Indian state of Jammu and Kashmir. The Sivalik Range is a mountain range that runs parallel to the Himalayas, along the northern border of India, Nepal, and Bhutan. The region is known for its rich biodiversity and unique forest ecosystems, including Himalayan dry temperate forests to subalpine forest types. It is home to a wide range of plant and animal species, including several that are endemic to the region (Figure 1). Using a species distribution model, we were able to identify the current locations where *Tragopan melanocephalus* is likely to be found (Figure 8). While *Tragopan melanocephalus* is known to occur in the Western Himalayan region spanning from northwest India through the Jammu and Kashmir area into Khyber Pakhtunkhwa in Pakistan, the distribution of suitable habitats across the upper Himalayas is uneven. Our findings reveal that areas with high and moderate suitability for the species are patchily distributed throughout the western Himalayas, ranging from northeastern Pakistan to central Himachal Pradesh and Uttarakhand. Moreover, there are continuous strips of highly suitable habitats along the Pakistan–Afghanistan border, in the Kashmir region of Uttarakhand, and in the Annapurna region. The analysis revealed that Kazinag National Park, Lachipora wildlife sanctuary, Tattakuti wildlife sanctuary, Limber wildlife sanctuary, and Khara Gali community reserve are the hotspots for *Tragopan melanocephalus* in the western Himalayan regions of India. Similarly, in Pakistan’s endemic region of *Tragopan melanocephalus*, the study identified core zones or hot places such as Indus Kohistan (Palas Valley), Kaghan Valley, Machiara National Park, Pir Chenasi, Nellum Valley, Salkhala, and Jugran Valley.

### 3.4. Suitability of Habitats under Future Scenarios of Climate Change

After examining the potential impacts of climate change on the species distribution, we found that the current most suitable habitat of *Tragopan melanocephalus* is likely to shrink and shift towards the north and across elevation gradients. According to the analysis of all four Shared Socioeconomic Pathways (SSPs) scenarios for the 2050s and 2070s, the predicted suitable habitat of *Tragopan melanocephalus* is expected to shift towards the north in Jammu and Kashmir, Pakistan (Khyber Pakhtunkhwa), and Uttarakhand, India. Consequently, there will likely be a contiguous range of highly suitable locations in the northern region of Jammu and Kashmir and its adjacent areas (Figure 8). Suitable habitats for *Tragopan melanocephalus* are projected to become increasingly scarce by 2050 under four different scenarios. In the SSPs 245 and SSPs 585 scenarios, the majority of accessible suitable locations will be in western Himachal Pradesh and Uttarakhand, as well as in the surrounding regions of Tibet near the border of the three countries. Under the SSPs 346 scenario, it is projected that a significant portion of the habitat between the Indian region and the Pak-Afghan border will disappear by 2050. Only a few narrow stretches of viable habitat are expected to remain in Himachal, specifically between the Kashmir region and Uttarakhand. According to the SSPs 245 and SSPs 585 scenarios, *Tragopan melanocephalus* may not have a suitable habitat in the Kashmir region, along the Pakistan–Afghanistan border, or in the Annapurna region by the year 2050.

Under all four-climate change scenarios examined, it is predicted that highly suitable habitats in the vicinity of the Pakistan-Afghanistan border, Kashmir, and Himachal will be lost by the 2070s. Additionally, the western Himalayan regions in Uttarakhand, spanning the Indian, Pakistani, and Afghan Himalayas, are also at risk of losing all their current suitable and highly suitable habitats by the 2050s and 2070s under the SSPs 245 and SSPs 585 scenarios. Moreover, between 2050 and 2070, only marginally suitable locations may be available in Pakistan, Kashmir, and central Himachal Pradesh, primarily in the Pak-Afghan border region.

**Figure 9:**
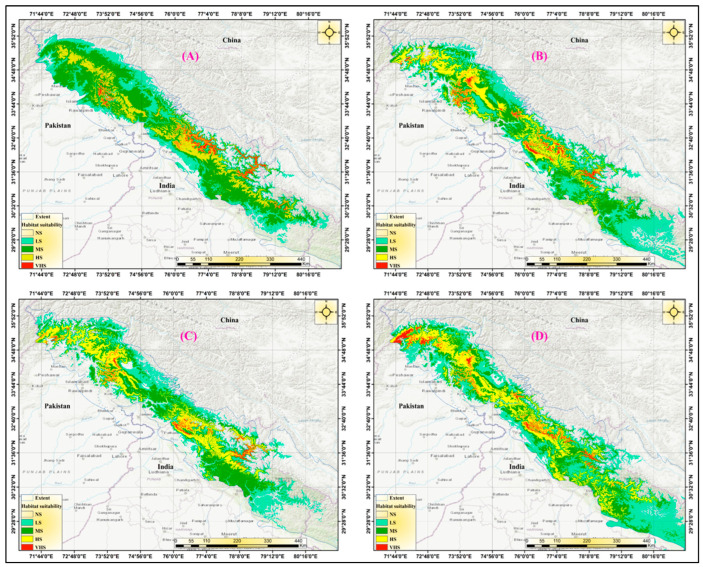
Maps generated by MaxEnt demonstrate the predicted habitat suitability classes under different future climate change scenarios. (**A**): SSPs-245 in the 2050s. (**B**): SSPs-585 in the 2070s. (**C**): SSPs-245 in the 2070s. (**D**): SSPs-585 in the 2070s.

A projection analysis revealed that the *Tragopan melanocephalus* habitat is anticipated to expand more under the SSPs 585 scenario by 2050, with a denser distribution over West Nepal, Uttarakhand, and their adjoining areas in Tibet compared to SSPs 245. However, by 2070, the habitat pattern would be similar in both scenarios. In SSPs 585, it is expected that *Tragopan melanocephalus* habitat would further expand in Uttarakhand and neighboring territories in Pakistan (Khyber Pakhtunkhwa). This prediction is illustrated in Figure 9.

According to this study, the total potential habitat suitability (*p* > 0.2) for Western Tragopan may decrease in the future. This decrease is estimated to be at a rate of −2.4% compared to the current distribution range, resulting in potential habitat suitability of 47,357 km^2^ under SSPs 245 of the 2050s, and a −3.7% rate of change, leading to potential habitat suitability of 41,045 km^2^ under SSPs 585 of 2050s. Similarly, under SSP 245 of 2070s, the potential habitat suitability is expected to reduce to 44,874 km^2^, which is a −2.9% rate of change, and 33,790 km^2^ (−5.2% rate of change) under SSPs 585 of 2070s. If we consider SSPs 245 in the 2050s, there is a prediction that the very high suitability habitat (VHS) will reduce slightly from its current climate, specifically from 9489 km^2^ to 7838 km^2^ (−0.3% rate of change). Similarly, within highly suitable habitat (VHS), the potentially suitable land area is estimated to reduce to 6156 km^2^ (−0.7%) under SSPs 585 of the 2050s, 6267 km^2^ (−0.6%) under SSPs 245 of 2070s, and 5358 km^2^ (−0.8%) under SSPs 585 of 2070s (Table 3). Due to projected climate change, there will be significant shifts in the suitable habitats within the Himalayan region of both Pakistan and India. In the Pakistani Himalayan part, the suitable habitat range is expected to shift from an elevation of 500 m to 800 m. Conversely, in the Indian Himalayan part, the suitable habitats will experience a shift from 600 m to 1000 m in elevation. These changes indicate the potential relocation of species and ecosystems as they adapt to the altered environmental conditions caused by climate change. The mean elevation shift of the habitat was 725 ± 221 m.

## 4. Discussion

The advancement of GIS technology in the last 20 years has facilitated the collection of spatial and temporal datasets, which has resulted in a more comprehensive understanding of species distribution patterns and the impact of environmental factors at various scales [53]. In this study, we present the first extensive analysis of *Tragopan melanocephalus’* distribution, habitat suitability, and projections of hotspots. The habitat suitability classes identified in this study (Table 3) indicate that areas with low suitability (probability 0.21–0.4), moderate suitability (probability 0.41–0.6), and high suitability (probability 0.61–0.8) for the Western Tragopan are expected to decrease, while regions with very high suitability (probability 0.81–1.0) are expected to expand significantly in the future. The increased building of dams, hydroelectric power plants, urbanization, and deforestation for agricultural purposes in the study area may be partially responsible for the accelerated rate of habitat fragmentation and degradation, which has resulted in the loss of acceptable habitats in specific locations [54,55].

The predictive ability of our model was robust, and it yielded highly significant results, as demonstrated by the AUC value exceeding 0.9 [56,57]. The AUC value of 0.992 for the current climate model indicated its precise and comprehensive characterization of *Tragopan melanocephalus’* habitat, accurately distinguishing between the presence and absence of the species in the western Himalayan region. Our assessment of habitat suitability was consistent with available occurrence records. In predicting the distribution of *Tragopan melanocephalus*, the model identified precipitation of the coldest quarter (bio19) and Mean Diurnal Range (Bio02) as the two most significant bioclimatic variables. This is congruent with independent studies [39,41] that identified annual precipitation (BIO 12) and mean diurnal range (Bio02) as the primary factors influencing the species distribution in the western Himalayan ranges of India and Pakistan. Notably, climate change in the western Himalayan regions of Pakistan has resulted in heavy precipitation and flooding in the valleys’ interiors, as recorded in 2008, 2010, 2017, 2020, and 2022.

The field-based observations of *Tragopan melanocephalus* presence records revealed that the species predominantly occupies elevations between 1850 to 3800 masl. This finding aligns with the species’ recognized inclination for extreme elevations, typically ranging from 1500 to 4000 masl. Several studies have provided supporting evidence regarding the preferred elevational range of *Tragopan melanocephalus*. Studies by Jameel et al. [54] and Awan et al. [39] conducted in the Western Himalayas (Pakistan) reported the presence of the species at elevations between 2000 and 4000 masl. Similarly, another study by Sing et al. [41] in the Western Himalayas (India) documented the species’ occurrence at elevations ranging from 1800 to 3800 masl. Our findings align with previous studies indicating that the preferred habitat for *Tragopan melanocephalus* in Pakistan and India is located in the upper mid-hill to high-mountain regions, specifically at elevations above 2000 masl [24,39,41]. This consistency in habitat preference across studies further strengthens the understanding of the species’ ecological requirements and provides valuable insights for conservation and management strategies in these regions. The species tends to favor areas with dense vegetation cover of 70–80% ground, including shrublands and forests. The common conifer trees in forest areas preferred by the species include *Pinus wallichiana, Abies pindrow, Picea smithiana,* and *Cedrus deodara,* along with another broad-leaved understory *vegetation* such as *Betula utilis, Quercus semecarpifolia, Acer caesium,* and *Juglans regia* [39,54]. However, we observed that the *Tragopan melanocephalus* is shifting further upward along the elevation and attempting to avoid the growing heat and anthropogenic disturbances during breeding time. Comparable findings were made by Awan et al. [39] who noted similar tree preferences for Western Tragopan in Pakistan. The projections from both SSPs 245 and 585 scenarios indicate that there will be an overlap in the habitat of *Tragopan melanocephalus* in the future, with a significant portion of the habitat shifting up to 725 ± 221 m from lowest range. These findings were supported by previous studies of Feeley et al. [58] which showed that montane forest habitats and species have migrated on average 6.1 m and Forero-Medina et al. [59] predicted a shift in their elevational range on average by around 500 m upslope per decade, respectively. The model identified two key bioclimate variables, namely, precipitation of the coldest quarter (bio19) and Mean Diurnal Range (Bio02), as the most influential factors in predicting the distribution of *Tragopan melanocephalus*. The model projections indicate that any changes in the species’ habitat along the elevation and aspect gradients in the study area are expected to be primarily longitudinal rather than latitudinal, suggesting that the species tends to be sedentary and remains within a specific home range throughout the year. Additionally, we observed that although *Tragopan melanocephalus* showed a general movement in their mean elevation, their elevational borders showed little change. The recurring pattern of an anticipated northward shift in *Tragopan melanocephalus* ranges in distinct Himalayan geographic regions offers strong proof that range shifts are a result of climate change [60].

The findings of this study are consistent with previous research [24,39,41,61], which suggests that aspect is a key factor in predicting the habitat of *Tragopan melanocephalus*, and agree with Jameel et al. [24] in identifying aspect as a distinguishing characteristic of this species’ habitat in the western Himalayas. Other studies in the western Himalayas of India and Pakistan also support the notion that the ideal habitat for Tragopan falls within a similar altitude range and is characterized by specific aspects [24,41,61]. Human activities, such as settlement patterns, may also play a role in habitat selection and directly impact the distribution of *Tragopan melanocephalus,* with evidence of habitat shrinkage due to new settlements in the study area, as also documented by Awan et al. [39] in their study.

Hunting and poaching were also identified as the most serious threats to the species’ current habitats. Our study supports the conclusions of the documented studies [24,39,41] that hunting poses major problems for the conservation of the Tragopan. The hunting season is most active in the winter when the species are more likely to be found close to human settlements. Hunting is most commonly done at night in Pakistan’s western Himalayan region and during the day in India’s western Himalayan region. During field surveys, it was also observed that the current habitat ranges of *Tragopan melanocephalus* are rapidly disturbed through non-timber forest collection, forest cutting, and overgrazing. Inskipp et al. [62] performed this type of analysis to determine the human pressures on threatened species, notably Western Tragopan, and discovered effects on distribution. We also noticed that the majority of the wildlife staff was illiterate, unequipped, and had no authority to deal with offenders. The same observations and findings were published by Awan et al. [39], who discovered that law enforcement authorities were weak and had little control over conservation issues, notably unlawful hunting.

Developing effective conservation strategies relies on understanding how species respond to climate change [63,64,65]. Research has shown that climate change has direct effects on various species and their ecosystems [63,66,67,68]. The primary objective of this study was to assess the potential changes in the distribution of *Tragopan melanocephalus* in response to various climate change scenarios. The MaxEnt modeling approach was used to predict the current and future distribution of the species in the western Himalayan region under various greenhouse gas emission scenarios over different periods. The study results indicate a projected decrease in the species’ distribution across the region in response to anticipated climate conditions for the years 2050 and 2070, as evaluated through the application of two selected SSPs (245 and 585). The simulations demonstrate the potential disappearance of the species, leading to a shift in both the overall range and core habitats in the future. Given its habitat confinement to isolated patches, *Tragopan melanocephalus* exhibits heightened vulnerability to climate change. This susceptibility is attributed to the species’ small ecological niche, which renders it more sensitive compared to species with broader ecological ranges [69,70]. This range shift may be due to the environmental envelope (precipitation and temperature) becoming less conducive to the species’ existence.

While climate change has a significant impact on determining the range of many species, it is important to acknowledge that other factors, such as dispersal patterns and capacity, ecological interactions, resource distribution and availability, and habitat preference, also contribute to their spatial distribution [31,71]. To achieve a more accurate understanding of the species’ range, these factors must be carefully studied and incorporated into species distribution models (SDMs) [45]. However, incomplete data and the challenge of fully integrating species ecology into modeling processes limit this work. Furthermore, factors such as sample size, sampling bias, predictor resolution, selection, and multicollinearity can impact SDM limitations and uncertainties and should be considered during modeling [72,73]. Despite these limitations, the MaxEnt modeling approach was used in this study, given its excellent predictive power and suitability for use with small sample sizes. While the study attempted to address issues such as multicollinearity and sampling bias, uncertainties in the results may still exist, as biotic interactions and dispersal capacity were not explicitly targeted. To improve the accuracy of future habitat distribution maps for the species, physiologically relevant parameters should be integrated into the modeling process. The study’s predictions for the region should be seriously considered for species conservation in the face of climate change, particularly global warming.

The local disappearance of species can lead to significant changes in ecosystem structure and function in any area [48,55,74]. Previous studies in several biodiversity hotspots with a high proportion of vulnerable species indicated that the risk of extinction has increased in recent years due to climate change, habitat degradation, and illegal hunting [23,41,75,76,77,78]. Our results are consistent with previous local or regional studies on climate in similar ecosystems and show that climate is a major factor influencing the habitat suitability of *Tragopan melanocephalus* [23,41]. Our study aimed to map the current and projected suitable habitats for *Tragopan melanocephalus* under different climate change scenarios and changing land use. To manage *Tragopan melanocephalus* and mitigate potential threats, it is essential to understand the impact of climate change and human threats on its distribution. By doing so, we also identified opportunities for protecting other species that share the habitat of *Tragopan melanocephalus*. The suitable location map and projections generated in this study can be used to identify critical habitat areas that require immediate conservation efforts to mitigate biodiversity loss due to habitat degradation and loss. The high-resolution maps generated by this study offer valuable support to park managers and conservationists by aiding in the identification and prioritization of conservation initiatives. These maps enable the identification of critical habitats at risk and the determination of appropriate conservation measures. By focusing on the preservation of threatened remnants and essential connecting habitats that are projected to vanish in the future, conservation efforts can be directed towards safeguarding these areas of utmost importance.

The present study is unique in that it examined the correlation between ecological variables and the livability of habitats, forecasts modifications in habitat suitability because of climate change, and models the distribution of *Tragopan melanocephalus* across its complete distributional range in the western Himalayas. Considering the scarcity of research on the distribution of *Tragopan melanocephalus* in the region, the findings from this study provide strong evidence for the need for further investigation. It is highly recommended that future investigations prioritize the predicted habitats that are projected to decline in the future. These habitats include regions such as Khyber Pakhtunkhwa in Pakistan and Uttarakhand in India. Additionally, it would be valuable to focus on monitoring the shifting suitable ranges in the coming years, particularly in the upper ridges of Kashmir and the areas adjacent to the Pakistan and Afghanistan borders. Conducting research in these specific areas will contribute to a better understanding of the species’ distribution patterns and will aid in conservation efforts. To promote conservation efforts, it is also recommended that the identified suitable areas be designated as nature reserves to support increased biodiversity and reduced risks under existing and anticipated future climatic conditions, and this study will help to formulate wildlife conservation management strategies.

### Implications for Conservation

Suitable habitat management and conservation directly influence and are the most important factors to reverse the loss of threatened wildlife taxa. Western Tragopan was recorded from unaltered, remote, and wilder habitats, and it was previously observed that the species is a bioindicator for the healthy habitat of the western Himalayan region. Therefore, we propose that this species be used as an indicator species in a western Himalayan ecological restoration project. This will increase conservation and protection for this flagship species. Studies like the current one should be applied to identify the future potential areas and such areas may be protected under community reserves, Protected Areas, and other Conservation Areas to ensure the long-term conservation of the species under the climate change scenario.

During field surveys, it was observed that the current habitat ranges of *Tragopan melanocephalus* are rapidly disturbed through non-timber forest collection, forest cutting, and overgrazing; as a result, we recommend that strategies be created to lessen the number or frequency of disturbances. Furthermore, large-scale infrastructure and power-generation projects have been observed to alter the habitat. As a result, we recommended that a biodiversity management plan be implemented before embarking on major infrastructure development projects. Poaching was identified as the second most serious threat to the species’ current habitats. The hunting season is most active in the winter when the species are most likely to be near human settlements. We observed that the wildlife staff was mostly illiterate, unequipped, and had little authority to combat the criminals. We suggested that the wildlife staff should be well equipped and knowledgeable, and have full authority to combat the criminals (illegal hunters and habitat destructors). Proactive measures can be taken to address potential hazards before they escalate into significant threats. Utilizing existing knowledge of suitable habitats in current and future can serve as a valuable guide for effective management. Our study findings revealed that the suitable overlap between the current and future predicted suitable habitat for *Tragopan melanocephalus* is primarily observed in the upper ridges of Waziristan, Upper Dir, and Chitral in Pakistan, as well as the northern region of Salkala and Jugran Valley in Kashmir. Consequently, we advised safeguarding these geographic centers of population connectedness in the future. The findings of this study suggest that a refined wildlife management strategy could significantly aid in the reintroduction of the *Tragopan melanocephalus* population in its western Himalayan suitable habitats, thus contributing to the global goals envisioned for the UN Decade (2021–2030) of Ecosystem Restoration targets.

## 5. Conclusions

This study emphasizes the significance of implementing conservation and habitat management strategies to safeguard endangered species, particularly the *Tragopan melanocephalus* located in the Himalayas’ western region. This species is a crucial indicator of the health of the western Himalayan region’s habitats and is threatened by habitat disturbance, hunting, and infrastructure development projects. This study used MaxEnt modeling to assess the species’ habitat suitability in varying climate scenarios in the western Himalayas and identified ten influential drivers. The study found that the habitat suitability of the Western Tragopan may increase under future climate change scenarios, but more research is necessary to prevent population collapses and identify other potential causes of local extinction events. The results can be used to create effective conservation plans for the species in a changing climate and serve as a basis for future monitoring of the species. Furthermore, the study provided field-based recommendations and implementation strategies to ensure the long-term survival of the targeted and other endangered species.

## Figures and Tables

**Table 1:** The environmental predictors used in the MaxEnt species distribution model (SDM) for *Tragopan melanocephalus* in the study area were as follows:

Data	Name of Variable & Description	Code	Resolution	Database
**Climatic Variables**	Annual Mean Temperature	Bio1	30 arc s	WorldClim
	Mean Diurnal Range	Bio2	30 arc s	WorldClim
	Temperature Seasonality (sd ×100)	Bio4	30 arc s	WorldClim
	Isothermality (Bio2/Bio7) (×100)	Bio3	30 arc s	WorldClim
	Mean Temperature of Wettest Quarter	Bio8	30 arc s	WorldClim
	Mean Temperature of Driest Quarter	Bio9	30 arc s	WorldClim
	Mean Temperature of Warmest Quarter	Bio10	30 arc s	WorldClim
	Mean Temperature of Coldest Quarter	Bio11	30 arc s	WorldClim
	Temperature Annual Range	Bio7	30 arc s	WorldClim
	Max. Temperature of Warmest Month	Bio5	30 arc s	WorldClim
	Min. Temperature of Coldest Month	Bio6	30 arc s	WorldClim
	Annual Precipitation	Bio12	30 arc s	WorldClim
	Precipitation of Wettest Month	Bio13	30 arc s	WorldClim
	Precipitation of Driest Month	Bio14	30 arc s	WorldClim
	Precipitation of Wettest Quarter	Bio16	30 arc s	WorldClim
	Precipitation of Driest Quarter	Bio17	30 arc s	WorldClim
	Precipitation Seasonality (CV)	Bio15	30 arc s	WorldClim
	Precipitation of Warmest Quarter	Bio18	30 arc s	WorldClim
	Precipitation of Coldest Quarter	Bio19	30 arc s	WorldClim
**Vegetation**	Enhanced Vegetation Index	EVI	30 arc s	Moderate Resolution Imaging Spectroradiometer MODIS/Terr
**Topographical** **Variables**	Elevation	Elev	30 arc s	SRTM DEM Global
	Aspect	Aspect	30 arc s	SRTM DEM Global
	Slope	Slope	30 arc s	SRTM DEM Global
**Anthropogenic** **Variables**	Human footprint	HFP	30 arc s	http://sedac.ciesin.columbia.edu (accessed on 2 June 2023)
	Road proximity	RP	30 arc s	http://sedac.ciesin.columbia.edu (accessed on 2 June 2023)
	Land cover	LC	30 arc s	http://www-modis.bu.edu/landcover (accessed on 2 June 2023)
	Population density	PD	30 arc s	http://www.ornl.gov/sci/landscan (accessed on 2 June 2023)

**Table 2:** Following initial data preprocessing, a subset of environmental, topographic, and anthropogenic variables was selected and evaluated for their respective contributions toward shaping ecological patterns.

Description	Code	Percent Contribution
Aspect	Aspect	36.5
Precipitation of Coldest Quarter	Bio19	34.7
Mean Diurnal Range	Bio02	13.3
Enhanced Vegetation Index	Evi	7.6
Precipitation of Driest Month	Bio14	5.2
Temperature Seasonality (sd × 100)	Bio04	1.7
Annual Precipitation	Bio12	0.4
Human Footprint	Hfp	0.4
Precipitation of Driest Quarter	Bio17	0.1
Temperature Annual Range	Bio07	0.1

**Table 3:** Expected probability of Western Tragopan habitat suitability under various climate change scenarios.

Climate Change Scenario	The Probability of Western Tragopan Occurrence within the Selected Habitat Suitability Categories Has Been Estimated					
	Not-Suitable (NS)	Least (LS)	Moderate (MS)	High (HS)	Very-High (VHS)	Total Suitable Land Area (km^2^)
	(*p* ≤ 0.2)	(*p* 0.21–0.4)	(*p* 0.41–0.6)	(*p* 0.61–0.8)	(*p* ≥ 0.81)	
**Current climate**	434,734	16,334	18,276	15,387	9489	59,486
**SSPs_245_2050**	446,863	12,778	13,778	12,963	7838	47,357
**Rate of change** (**%**)	2.45	−0.72	−0.91	−0.49	−0.33	−2.45
**SSPs_585_2050**	453,175	11,234	12,422	11,233	6156	41,045
**Rate of change** (**%**)	3.7	−1.0	−1.2	−0.8	−0.7	−3.7
**SSPs_245_2070**	449,346	12,345	14,267	11,995	6267	44,874
**Rate of change** (**%**)	2.96	−0.81	−0.81	−0.69	−0.65	−2.96
**SSPs_585_2070**	460,430	9027	9138	10,267	5358	33,790
**Rate of change** (**%**)	5.20	−1.48	−1.85	−1.04	−0.84	−5.20

## Data Availability

This publication contains all the available data.
